# Individual-level determinants of depressive symptoms and associated diseases history in Turkish persons aged 15 years and older: A population-based study

**DOI:** 10.3389/fpsyt.2022.983817

**Published:** 2022-12-01

**Authors:** Yohane V. A. Phiri, Kemal Aydın, Nadire Gülçin Yıldız, Mfundi President Sebenele Motsa, Owen Nkoka, Halide Z. Aydin, Hsing Jasmine Chao

**Affiliations:** ^1^School of Public Health, College of Public Health, Taipei Medical University, Taipei, Taiwan; ^2^Institute for Health Research and Communication, Lilongwe, Malawi; ^3^Faculty of Economics and Administrative Sciences, Amasya University, Amasya, Turkey; ^4^Department of Guidance and Counseling, Faculty of Education, Istanbul Medipol University, Istanbul, Turkey; ^5^Global Health and Health Security, College of Public Health, Taipei Medical University, Taipei, Taiwan; ^6^Behavioural Research and Innovations Unit, Educational Youth Empowerment, Manzini, Eswatini; ^7^Institute of Health and Wellbeing, University of Glasgow, Glasgow, United Kingdom; ^8^Arnold School of Public Health, University of South Carolina, Columbia, SC, United States; ^9^Neuroscience Research Center, Taipei Medical University, Taipei, Taiwan

**Keywords:** depressive symptoms, patient health questionnaire-8, chronic conditions, Turkey health survey, individual determinants

## Abstract

**Background:**

Depressive symptoms are associated with both long-lasting and short-term repetitive mood disorders and affect a person’s ability to function and lead a rewarding life. In addition to predisposing genetic causes, other factors such as socioeconomic and demographic factors, and chronic diseases have also been reported to associate with depression. In this study, we analyzed the association between history of chronic diseases and presentation of depressive symptoms amongst Turkish individuals.

**Methods:**

We employed the 2019 Turkey health survey to analyze data of 11,993 individuals aged 15+ years. Depressive symptoms were assessed using the eight-item Patient Health Questionnaire (PHQ-8) coded with a binary measure, a score of <10 as less depressed and >10 as moderate-severely depressed. A number of sociodemographic characteristics were adjusted for in the analyses. Logistic regression models were used to test the association between chronic diseases and depressive symptoms in the study sample.

**Results:**

Our analysis revealed that 6.24% of the 11,993 participants had reported an episode of depressive symptoms. The prevalence of depressive symptoms in men was 1.85% and in women, it was 2.34 times higher. Participants who had previously reported experiencing coronary heart diseases (AOR = 7.79, 95% CI [4.96–12.23]), urinary incontinences (AOR = 7.90, 95% CI [4.93–12.66]), and liver cirrhosis (AOR = 7.50, 95% CI [4.90–10.42]) were approximately eight times likely to have depressive symptoms. Similarly, participants with Alzheimer’s disease (AOR = 6.83, 95% CI [5.11–8.42]), kidney problems (AOR = 6.63, 95% CI [4.05–10.85]), and history of allergies (AOR = 6.35, 95% CI [4.28–9.23]) had approximately seven-fold odds of reporting episodes of depressive symptoms. The odds of presenting with depressive symptoms amongst participants aged ≥ 50 were higher than in individuals aged ≤ 49 years.

**Conclusion:**

At individual level, gender and general health status were associated with increased odds of depression. Furthermore, a history of any of the chronic diseases, irrespective of age, was a positive predictor of depression in our study population. Our findings could help to serve as a reference for monitoring depression amongst individuals with chronic conditions, planning health resources and developing preventive and screening strategies targeting those exposed to predisposing factors.

## Introduction

Depression is a mental health disorder characterized by symptoms of low mood and energy, which affects people’s thoughts, feelings, behaviors, and sense of well-being. Depression is a leading cause of disability and contributes significantly to the global burden of diseases (1). Depressive symptoms are both long-lasting and short-term repetitive mood disorders and affect a person’s ability to function and lead a rewarding life. This common psychological condition affects nearly 350 million people of all age groups globally (2–4). The exact cause of depression is unknown and literature indicates that it may be caused by a combination of genetic, environmental, biological, and psychological factors (5). In addition to predisposing genetic causes, other factors such as socioeconomic and demographic factors (6), chronic diseases have also been reported to be associated with depression (7). Recent findings have shown that in addition to the previous mentioned factors, depressive symptoms are linked to behavioral risk factors such as obesity (8), physical inactivity (9), and alcohol and tobacco use (10). Studies on the etiology and exacerbation of depression have concluded that the negative interaction and timing of the various predictors associated with depressive symptoms in individuals is essential (11).

In Turkey, research on depressive symptoms has been conducted with a focus on special populations (pregnant women, the elderly, university students, and health workers) (12). To our knowledge, studies generalizable to the country’s population are limited. The most recent survey generalizable to the Turkish population was conducted between 1995 and 1996 (13). There are several changes that might have occurred and these are irrefutably positively or negatively associated with mental health and wellbeing of the Turkish population. According to the findings presented by Kılıç et al. (13), the first epidemiological study conducted in Turkey on mental health disorders, 17.2% of the Turkish population had mental illnesses. Furthermore, the study indicated that the prevalence of depressive symptoms in women was two times higher than in men. The cause for the observed difference in the prevalence of depressive symptoms between men and women have also been documented elsewhere (14, 15). Some of the factors include: the interaction between observable social and health inequalities and sociodemographic factors (14, 16) and hormonal role in women (15, 17).

A study employing 2013 survey conducted by the ministry of health (MoH) in Turkey on the risk factors of chronic diseases found that the prevalence of depression amongst patients with chronic conditions was high (18). The study reported that depressive symptoms had significant association with various chronic conditions such panic disorders, chronic asthma symptoms, and cardiovascular diseases. It has also been reported that amongst Turkish elderly population, chronic diseases and gender (being a woman) are significant predictors of depressive symptoms (19). A systematic review conducted in several developing and emerging countries further revealed that chronic diseases (diabetes, obesity, cancer, heart disease, and COPD) are significant predictors of the presentation of depressive symptoms, anxiety, bipolar disorders, and schizophrenia across populations of different age groups (20). Various medical chronic conditions have been associated with a higher prevalence of depressive symptoms (21–23). Specifically, depressive symptoms in those with chronic illnesses is perceived to originate from either the biological effect of chronic diseases (i.e., diseases affecting the central nervous systems such as Parkinson’s disease, cerebrovascular disease, or multiple sclerosis) or behavior mechanisms mediated by limitations imposed by chronic illnesses.

The European Health Interview Survey (EHIS), has been conducted periodically since 2006 in 27 European Union (EU) countries, the United Kingdom, Norway, Iceland, and Turkey (24). In Turkey, EHIS is translated as the Turkey Health Survey (THS) with modification of the EHIS protocol to suit the Turkish population (25). In Turkey, literature employing PHQ-8 to assess population depressive symptoms is limited despite a number of studies within the EU employing the screening tool in population surveys (26–31). In this article, we aim to analyze the microdata of the THS conducted in 2019 to establish the prevalence of depression and depressive symptoms in the adult population aged 15+ years in Turkey. We further examine how depressive symptoms are associated with individual-level factors and chronic diseases.

## Materials and methods

### Study design, sampling, and data collection

Turkey has a population of around 84,680,273 with a median age of 33.1 years and 9.7% of the elderly aged 65+ years (32). For this cross-sectional study we employed the Turkey Health Survey (THS) data, a part of the European Health Interview Survey Wave 3 (EHIS-3) which is conducted in all European member states with the exception of some territories within the United Kingdom (UK), France, Netherlands, Ireland, and Cyprus (24). The EHIS3 is designed to harmonize and present a tool for comparison amongst member states on the health status, health determinants, use of health care services and limitation to access of their citizens. The THS employed in this study had a total 23,199 participants targeting the whole population with 17,084 participants aged 15 years and above. Our analysis employed 11,993 individuals of both sexes aged 15+ years who had complete information on both sociodemographic characteristics and PHQ-8 questionnaire. The THS included participants aged 15+ years as eligible for completion of self-reported depressive symptoms questionnaire, in addition to all other variables considered in the survey. We targeted a population-based study hence the inclusion of all the participants who had complete data inclusive of individuals ≥15 years old.

Details relating to the full methodology employed in the study are described elsewhere (24, 25). In brief the European Health Interview Survey (EHIS) was developed between 2003 and 2006, comprising of four modules covering variables on citizens health status, health determinants, use of health care services and limitation of access and social demographic characteristic of the population. Currently, three waves of the EHIS have been conducted in different member states of the European Union (EU), the United Kingdom, Norway, Iceland, and Turkey. The first EHIS was conducted between 2008 and 2009 in 17 EU states, followed by wave 2 conducted between 2013 and 2015 in all EU member states and the third wave in 2019. The EHIS employs the same four modules in each member state, with each of the country allowed to modify the questions and frequency of the period of conducting the survey to satisfy their country needs within each module.

The THS was conducted by Turkish Statistical Institute employing instruments provided by EHIS. Previously, upon inception of the EHIS in 2008, until 2016 THS was conducted every 2 years. The data used in this study was collected in 2019 across 12 regions to represent the population of Turkey in the 81 provinces and 957 districts of Turkey. The selection of the survey sample employed a multi-stage probability clustering sampling technique. All persons aged 15+ years were eligible to participate in the survey with inclusion of their province being a prerequisite during the sampling stage. Computer assisted personal interviews was employed to collect the data. Face to face interviews were conducted by trained interviewers, completing questionnaires with each of the participants enrolled in the study.

The THS questionnaire was structured to obtain data on background variables (socio-demographic data), health status, health determinants, access to healthcare services and limitations associated. In this study the data employed comprised of self-reported responses of all the participants enrolled. The data extracted for use asked the participants of their social demographic characteristics (age, marital status, education, physical activity level, general health status, etc.), history of diseases condition of each of the participants within the past 12 months (Asthma, bronchitis, experience of allergic reactions, etc.) and level of depressive symptoms within the past 2 weeks assessed using patient health questionnaire (PHQ-8). Anonymized microdata is provided by the Turkish Statistical Institute after signing an agreement regarding the security, confidentiality, accessibility, and use of the data.

### Study variables

#### Outcome variables

Our outcome variable was episodes of depressive symptoms amongst individuals aged 15+ years assessed through PHQ-8 questionnaire. In order to quantify depressive symptoms status at individual level, participants were required to complete eight questions in the PHQ-8 each scaling 0–3 (where 0 = Not at all, 1 = Several days, 2 = More than half the days, and 3 = Nearly every day.) with a total severity score ranging 0–24. The PHQ-8 which was part of the THS includes questions which read; Over the last 2 weeks, how often have you been bothered by any of the following Y things (where Y is one of the eight questions in the PHQ-8 given). The questions included in the PHQ-8 include: (1) “Little interest or pleasure in doing things,” (2) “Feeling down, depressed, or hopeless,” (3) “Trouble falling or staying asleep, or sleeping too much,” (4) “Feeling tired or having little energy,” (5) “Poor appetite or overeating,” (6) “Feeling bad about yourself—or that you are a failure or have let yourself or your family down,” (7) “Trouble concentrating on things, such as reading the newspaper or watching television,” and (8) “Moving or speaking so slowly that other people could have noticed? Or the opposite—being so fidgety or restless that you have been moving around a lot more than usual.” A binary measure of the computed total depressive symptoms severity was coded 0 (i.e., a score < 10 as no or less depression) or 1 (i.e., a score ≥ 10 as moderate-severely depressed) (33). The Turkish version of the PHQ-8 has been evaluated to be reliable and valid in a number of studies conducted amongst Turkish populations with various chronic illnesses (34, 35).

#### Primary independent variables

The primary independent variable of our study was history of chronic diseases amongst the study participants in the THS. During the survey, individuals aged 15+ years were asked whether they had experienced any chronic diseases within the past 12 months (24, 25). The responses were self-reported based on the respondent’s history of diagnosis of the chronic diseases. Each of the chronic diseases variable was coded “Yes” (referring to participants with a history of diagnosis) and “No” (for those without a history of diagnosis). The participants were asked, “*during the past 12 months, have you had or were you diagnosed with any of the following chronic diseases”:* asthma or allergic asthma, chronic bronchitis (chronic obstructive pulmonary disease or emphysema), myocardial infarction (heart attack) or chronic consequences of myocardial infraction, coronary heart disease or angina pectoris, high blood pressure (hypertension), stroke (cerebral hemorrhage, cerebral thrombosis) or chronic consequences of stroke, arthrosis (arthritis excluded), low back disorder or other chronic back defect, neck disorder or other chronic neck defect, diabetes mellitus, any allergy such as rhinitis, hay fever, eye inflammation dermatitis, food allergy or other allergy (allergic asthma excluded), cirrhosis of the liver, urinary incontinence or problems in controlling the bladder, kidney problems, high blood lipids (high cholesterol and triglyceride), and Alzheimer’s disease.

#### Sociodemographic characteristics

All variables considered as sociodemographic characteristics in the study were selected based on literature (36, 37). We included variables gender (Male or Female), age of the participants (15–30, 31–50, 51–70, or >70), marital status (Single, Married, Widowed, or Divorced), education level (Primary and below, Secondary School, High School, or College and above), employment status (Unemployed, Continuing Education, or Employed), obesity (Normal Weight, Pre-obese Stage, or Obese), physical activity (No activity, Mild Activity, Moderate Activity, or Intense Activity), and general health status (Good, Fair, or Bad).

### Statistical analyses

We examined the distribution of the study characteristics and diseases condition by depressive symptoms status. Binary logistic regression was employed to measure the association between the outcome of interest and the independent variables. We did run a stepwise regression model for the covariates to determine their association with depressive symptoms status. All sociodemographic characteristics which remained significantly associated with depressive symptoms in the stepwise regression approach were adjusted in the multivariable models. All sociodemographic characteristics adjusted in the multivariable models had a *p*-value < 0.1 (38). We tested correlation of the variables included in both the stepwise and multivariable regression model using a correlation matrix function in SAS. All variables with correlation value of ≥0.8 were dropped from the models (39, 40). Evidence demonstrates that depressive symptoms in late-life (≥50-year-old) are significantly associated with several medical morbidities (36, 37, 41). This observation vindicates the complex relationship between mental health and physical health amongst a healthy elderly population. Hence, we further conducted a stratified analysis of depressive symptoms in our study sample by age (≤49 years vs. ≥ 50 years). Additionally, all variable were added into the regression model one by one whilst assessing the stability of the models. Results were reported as adjusted odd ratio (AOR) with their 95% confidence intervals as a measure of association between the outcome and independent variables. All analyses were run using SAS 9.4 (SAS Institute Inc., Cary, NC, USA) and the statistical significance was set at *p* < 0.05.

### Ethical approval

The study protocol for the THS was approved by the chairperson of the Turkish Statistical Institute. “Regulation on Procedures and Principles Regarding Confidential Data Privacy and Data Security in the Official Statistics” were employed to ensure privacy and confidentiality of the data from the study. These guidelines were officially published and a gazette assented to on 20/06/2006-No.26204. All participants provided informed consent before enrollment into the study.

## Results

### Prevalence of chronic diseases

**FIGURE 1:**
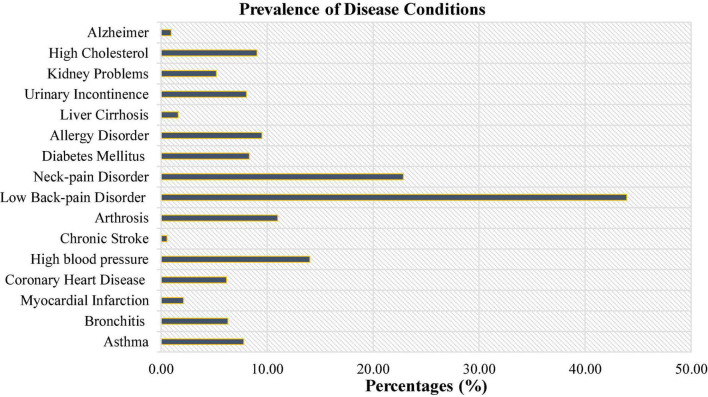
Prevalence of the different types of disease condition among the study participants.

Our study recruited a total of 11,993 participants and data for those who did not take part in the study or provided incomplete information was treated as missing. Figure 1 below shows the prevalence of diseases reported by the participants included in this study. The results indicated that of the surveyed participants, 43.93% reported of lower back pain and was the highest of all diseases condition. The lowest disease condition reported amongst our study participants was chronic consequences of stroke (0.54%).

### Study characteristics by episodes of depressive symptoms

The results in Table 1 showed that 6.24% (*n* = 748) of the surveyed participants had an episode of depressive symptoms. We observed significant differences (*p* < 0.05) between the various individual factors considered and episodes of depressive symptoms. Participants who were married (62.17%), had only completed primary school level/below of education (67.78%), did not partake in any form of physical activity (34.76%) and reported a poor general health condition (43.32%) had a higher prevalence of episodes of depressive symptoms. We did observe a nearly uniform distribution of episodes of depressive symptoms amongst participants within the obesity group (Not obese, Pre-obese, and Obese).

**TABLE 1:** Distribution of study characteristics by depressive symptoms.

Variable	Depressive symptoms				UOR (95% CI)	*P*-value^a^
	Yes		No			
	*n* = 748	%	*n* = 11,245	%		
**Gender**						
Male	222	29.68	5,276	46.92	Ref	Ref
Female	526	67.21	5,969	53.08	2.09 [1.78–2.46]	**<0.0001**
**Age group**						
15–30	118	15.78	3,457	30.74	Ref	Ref
31–50	246	32.89	4,504	40.05	1.60 [1.28–2.00]	**0.011**
51–70	243	32.49	2,655	23.61	2.68 [2.14–3.36]	**<0.0001**
>70	141	18.85	629	5.59	6.57 [5.07–8.51]	**<0.0001**
**Marital status**						
Single	99	13.24	2,711	24.11	Ref	Ref
Married	465	62.17	7,589	67.49	1.67 [1.35–2.09]	**<0.0001**
Widowed	39	5.21	339	3.01	3.15 [2.14–4.64]	**<0.0001**
Divorced	145	19.39	606	5.39	6.55 [5.00–8.59]	**<0.0001**
**Education level**						
Primary and below	507	67.78	4,686	41.67	Ref	Ref
Secondary school	93	12.43	2,151	19.13	0.40 [0.32–0.50]	**<0.0001**
High school	83	11.1	2,274	20.22	0.34 [0.27–0.43]	**<0.0001**
College and above	65	8.69	2,134	18.98	0.28 [0.22–0.37]	**<0.0001**
**Working status**						
Unemployed	52	6.95	704	6.26	Ref	Ref
Continuing education	25	3.34	999	8.88	0.34 [0.21–0.55]	**<0.0001**
Employed	671	89.71	9,542	84.86	0.95 [0.71–1.28]	0.7417
**Obesity**						
Normal weight	260	34.76	4,947	43.99	Ref	Ref
Pre-obese stage	246	32.89	4,009	35.65	1.17 [0.98–1.40]	**<0.0001**
Obese	242	32.35	2,289	20.36	2.01 [1.68–2.41]	**<0.0001**
**Physical activity**						
No activity	255	34.09	1,641	14.59	Ref	Ref
Mild activity	299	39.97	4,758	42.31	0.40 [0.34–0.48]	**<0.0001**
Moderate activity	158	21.12	3,783	33.64	0.27 [0.22–0.33]	**<0.0001**
Intense activity	36	4.81	1,063	9.45	0.22 [0.15–0.31]	**<0.0001**
**General health status**						
Good	166	22.19	7,655	68.07	Ref	Ref
Fair	258	34.49	2,727	24.25	4.36 [3.57–5.33]	**<0.0001**
Poor	324	43.32	863	7.67	17.3 [14.1–21.1]	**<0.0001**

UOR, unadjusted odds ratio; 95% CI, 95% confidence interval.

^a^*P*-value for regression analyses, bold means significant, i.e., *p* < 0.05.

### Diseases condition by episodes of depressive symptoms

Table 2 shows the distribution of chronic diseases by episodes of depressive symptoms. The results revealed that participants who had a history of neck disorders (*n* = 429), lower back pain disorders (*n* = 643), high blood pressure (*n* = 305), and arthrosis (*n* = 254) had higher prevalence of episodes of depressive symptoms. Furthermore, those who had asthma (62.5%), experienced allergy disorders (65.12%) and reported a high cholesterol level (68.28%) had higher than 50% prevalence of episodes of depressive symptoms.

**TABLE 2:** Distribution of diseases conditions by depressive symptoms.

Variable	Depressive symptoms				UOR (95% CI)	*P*-value^a^
	Yes		No			
	*n* = 748	%	*n* = 11,245	%		
**Asthma**						
Yes	175	62.5	759	10.29	14.5 [11.3–18.7]	**<0.0001**
No	105	37.5	6,619	89.71	ref	
**Bronchitis**						
Yes	164	60.97	590	8.18	17.5 [13.5–22.7]	**<0.0001**
No	105	39.03	6,619	91.82	ref	
**Myocardial infarction**						
Yes	84	44.44	168	2.48	31.5 [22.8–43.6]	**<0.0001**
No	105	55.56	6,619	97.52	ref	
**Coronary heart disease**						
Yes	199	65.46	542	7.57	23.1 [18.0–29.8]	**<0.0001**
No	105	34.54	6,619	92.43	ref	
**High blood pressure**						
Yes	305	74.26	1,379	17.24	13.9 [11.0–17.4]	**<0.0001**
No	105	25.74	6,619	82.76	ref	
**Chronic stroke**						
Yes	28	21.05	37	0.56	4.71 [2.9–8.8]	**<0.0001**
No	105	78.95	6,619	99.44	ref	
**Arthrosis**						
Yes	254	71.75	1,066	13.87	15.0 [11.9–19.0]	**<0.0001**
No	105	29.25	6,619	86.13	ref	
**Low back disorder**						
Yes	643	85.96	4,626	41.14	8.76 [7.1–10.8]	**<0.0001**
No	105	14.04	6,619	58.86	ref	
**Neck disorder**						
Yes	429	80.34	2,311	25.88	11.7 [9.4–14.6]	**<0.0001**
No	105	19.66	6,619	74.12	ref	
**Diabetes mellitus**						
Yes	192	64.65	807	10.87	14.9 [11.7–19.2]	**<0.0001**
No	105	35.35	6,619	89.13	ref	
**Allergy disorder**						
Yes	196	65.12	945	12.49	13.1 [10.2–16.7]	**<0.0001**
No	105	34.88	6,619	87.51	ref	
**Liver cirrhosis**						
Yes	50	32.26	145	2.14	21.7 [14.9–31.6]	**<0.0001**
No	105	67.74	6,619	97.86	ref	
**Urinary incontinence**						
Yes	220	67.69	747	10.14	18.6 [14.5–23.7]	**<0.0001**
No	105	32.31	6,619	89.86	ref	
**Kidney problems**						
Yes	142	57.49	485	6.83	18.5 [14.1–24.1]	**<0.0001**
No	105	42.51	6,619	93.17	ref	
**High cholesterol**						
Yes	226	68.28	857	11.46	16.6 [13.0–21.2]	**<0.0001**
No	105	31.72	6,619	88.54	ref	
**Alzheimer’s disease**						
Yes	45	30	71	1.06	9.96 [6.2–10.8]	**<0.0001**
No	105	70	6,619	98.94	ref	

UOR, unadjusted odds ratio; 95% CI, 95% confidence interval.

^a^*P*-value for regression analyses, bold means significant, i.e., *p* < 0.05.

### Association between individual level factors and episodes of depressive symptom

Univariate regression analyses for individual level factors showed that all the individual level factors were associated with episodes of depressive symptoms amongst the study participants (Table 1). A stepwise regression analyses of the individual variables (all individual level variables included) revealed that women had a higher likelihood of reporting episodes of depressive symptoms (AOR = 1.30, 95% CI [1.08–1.56]) as compared to men. Furthermore, participants who reported either having a fair (AOR = 4.31, 95% CI [3.45–5.38]) or poor (AOR = 15.29, 95% CI [11.96–19.56]) general health status were more likely to have depressive symptoms. On the other hand, we did observe that working status and physical activity were negatively associated with reported episodes of depressive symptoms. Participants who reported continuing their education (AOR = 0.49, 95% CI [0.29–0.82]) or being employed (AOR = 0.53, 95% CI [0.38–0.75]) were less likely to report episode of depressive symptoms compared to participants not employed (see Table 3).

**TABLE 3:** Multivariate regression model for individual level factors.*

Variables	AOR (95% CI)^#^	*P*-value^a^
**Gender**		
Male	Ref	
Female	1.30 [1.08–1.56]	**0.005**
**Age group**		
15–30	Ref	
31–50	1.00 [0.74–1.35]	0.9829
51–70	0.81 [0.58–1.12]	0.203
>70	0.84 [0.57–1.24]	0.378
**Marital status**		
Single	Ref	
Married	0.85 [0.61–1.17]	0.3057
Widowed	1.54 [0.96–2.46]	0.0721
Divorced	1.32 [0.88–1.97]	0.1857
**Education level**		
Primary and below	Ref	
Secondary school	1.096 [0.785–1.530]	0.5907
High school	0.923 [0.662–1.285]	0.6339
College and above	0.779 [0.547–1.110]	0.1667
**Working status**		
Unemployed	Ref	
Continuing education	0.49 [0.29–0.82]	**0.007**
Employed	0.53 [0.38–0.75]	**0.000**
**Obesity**		
Normal weight	Ref	
Pre-obese stage	0.91 [0.75–1.11]	0.3529
Obese	1.02 [0.83–1.26]	0.8433
**Physical activity**		
No activity	Ref	
Mild activity	0.59 [0.49–0.72]	** < 0.0001**
Moderate activity	0.58 [0.46–0.74]	** < 0.0001**
Intense activity	0.57 [0.39–0.84]	**0.004**
**General health status**		
Good	Ref	
Fair	4.31 [3.45–5.38]	** < 0.0001**
Bad	15.29 [11.96–19.56]	** < 0.0001**

*Variables added into the regression model using a stepwise approach. Ref, reference group for each variable in the logistic regression analyses.

^#^AOR, adjusted odds ratio; 95% CI, 95% confidence interval.

^a^*P*-value for regression analyses, bold means significant, i.e., *p* < 0.05.

### Association between chronic diseases and episodes of depressive symptom

**FIGURE 2:**
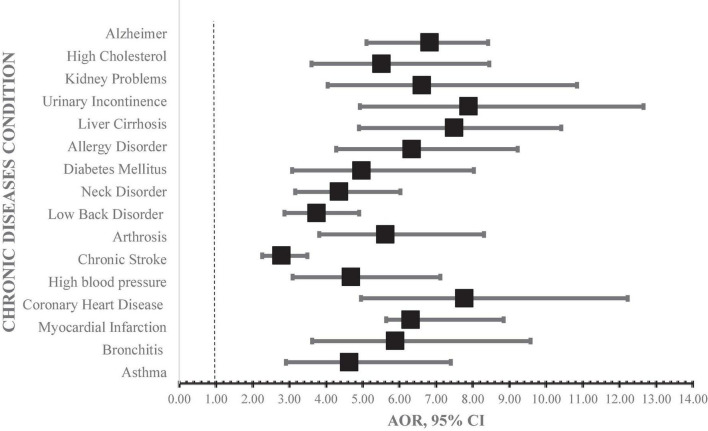
Multivariate regression models for history of chronic diseases. AOR, adjusted odds ratio; 95% CI, 95% confidence interval. Model adjusted for individual level factors (gender, employment status, physical activity, general health status).

Figure 2 below is forest plot of the odds of depressive symptoms associated with the reported chronic diseases in our study sample. The results revealed that all of the reported chronic diseases were significantly associated with higher odds of episodes of depressive symptoms. Participants who had previously reported experiencing coronary heart diseases (AOR = 7.79, 95% CI [4.96–12.23]), urinary incontinences (AOR = 7.90, 95% CI [4.93–12.66]), and liver cirrhosis (AOR = 7.50, 95% CI [4.90–10.42]) were 7.5 times likely to have depressive symptoms. Similarly, participants with Alzheimer’s disease (AOR = 6.83, 95% CI [5.11–8.42]), kidney problems (AOR = 6.63, 95% CI [4.05–10.85]), and history of allergies (AOR = 6.35, 95% CI [4.28–9.23]) had approximately seven-fold odds of reporting episodes of depressive symptoms.

Furthermore, the results revealed that both individuals aged ≤49 years and ≥50 years had higher odds of presenting with depressive symptoms if they had reported a history of any of the chronic disease in the study. However, the odds of presenting with depressive symptoms amongst participants aged ≥ 50 were higher compared to individuals aged ≤ 49 years (Table 4).

**TABLE 4:** Multivariate regression models for history of chronic diseases by age.*

Variables	Age groups							
	≤49 years (*n* = 8,123)				≥50 years (*n* = 3,870)			
	AOR	95% CI		*P*-value^a^	AOR	95% CI		*P*-value^a^
Asthma	5.24	3.05	8.99	**<0.0001**	9.00	3.12	10.97	**<0.0001**
Bronchitis	5.39	3.05	9.51	**<0.0001**	6.95	5.49	9.39	**<0.0001**
Myocardial infarction	5.69	4.39	6.99	**<0.0001**	7.71	6.10	8.22	**<0.0001**
Coronary heart disease	7.12	4.08	12.45	**<0.0001**	7.91	5.96	8.19	**<0.0001**
High blood pressure	6.65	3.92	11.26	**<0.0001**	7.13	3.23	7.97	**<0.0001**
Chronic stroke	1.40	1.27	7.54	**<0.0001**	5.24	4.51	9.76	**<0.0001**
Arthrosis	6.24	3.96	9.86	**<0.0001**	8.33	5.46	10.01	**<0.0001**
Low back disorder	3.73	2.78	5.00	**<0.0001**	5.90	2.75	7.66	**<0.0001**
Neck disorder	4.37	3.03	6.29	**<0.0001**	7.25	3.22	10.33	**<0.0001**
Diabetes mellitus	4.01	2.09	7.71	**<0.0001**	6.96	4.56	9.37	**<0.0001**
Allergy disorder	6.58	4.29	10.09	**<0.0001**	8.33	3.39	10.44	**<0.0001**
Liver cirrhosis	6.97	2.90	9.73	**<0.0001**	7.03	5.65	10.51	**<0.0001**
Urinary incontinence	11.06	5.96	20.52	**<0.0001**	13.12	5.21	18.09	**<0.0001**
Kidney problems	5.86	3.21	10.68	**<0.0001**	8.53	6.14	9.25	**<0.0001**
High cholesterol	5.94	3.42	10.32	**<0.0001**	9.59	3.91	13.50	**<0.0001**
Alzheimer’s disease	5.20	3.67	6.13	**<0.0001**	6.31	3.64	7.51	**<0.0001**

AOR, adjusted odds ratio; 95% CI, 95% confidence interval.

*Model adjusted for individual level factors (gender, employment status, physical activity, general health status).

^a^*P*-value for regression analyses, bold means significant, i.e., *p* < 0.05.

## Discussion

In this large national representative cross-sectional survey of a well-characterized sample of 11,993 individuals aged 15 and above across Turkey, we did observe that depressive symptoms were significantly associated with history of diagnosis of various chronic diseases. Our study also revealed that gender, general health status, physical activity, and work status were individual level factors significantly associated with depressive symptoms. Furthermore, irrespective of age of the study participants, depressive symptoms were significantly associated with those we grouped as older (≥50 years) and young persons (≤49 years). Our findings provide further evidence for a strong relationship between depressive symptoms and history of chronic diseases status in a population.

Our results demonstrate that the current prevalence of depressive symptoms amongst the Turkish population was 6.24% and women were twice likely to present with depressive symptoms irrespective of their age. These results concur with past and recent epidemiological studies conducted both in Turkey and other EU states. A study conducted by the MoH in Turkey in 1998, reported a 4% population prevalence of depressive symptoms with women having a higher prevalence of 5.4% (13). Additionally, recent studies conducted amongst populations of different age groups have shown the prevalence of depressive symptoms to be as high as 9% in Turkey (12). Amongst Turkish adolescents, the prevalence of depressive symptoms has been pegged between 9 and 12.5% (42, 43) while in adults it varies according to age and other predisposing factors such as chronic diseases (44). Studies conducted in several countries in the EU, employing the EHIS, have also reported high prevalence of depressive symptoms. In the UK, age and sex were determined as significant predictors of depressive symptoms amongst individuals aged 15+ years with a prevalence of 11.3% (28). Similarly, studies conducted in Spain and Serbia reported a respective 8 and 10% prevalence of depressive symptoms (26, 31).

This study also revealed a high prevalence of depressive symptoms amongst those with a history of diagnosis of low back-pain and neck-pain disorders. Depression, chronic neck-pain, and back-pain disorders have been reported to be the most common encountered health conditions in the general population (45). Increase in the prevalence of depressive symptoms amongst individuals with chronic pain in any location of their body has been reported (46). A study conducted in the EU Countries (Spain, Germany, Italy, and Portugal) and the UK showed that 43.4% of individuals with a history of painful physical conditions were four times likely to present with major depressive episodes (47). Similarly, a longitudinal study conducted in Spain reported an association between a higher prevalence of low back pain amongst men (24.7%) and women (38.6%) and symptoms of depressive symptoms (48). The etiology of chronic neck and back-pain disorders is multifactorial, and hence the association with depressive symptoms in individuals is bidirectional. Earlier studies have shown back and neck-pain as a composite outcome of various biopsychosocial factors such age, depression and comorbidities (45, 46). Conversely, individuals with chronic back and neck-pain disorders are likely to experience discomfort and hence at a greater risk of depression (45, 47, 49). Thus, either depression or chronic low back-pain and neck disorders may be the cause of the other.

We did also observe that amongst the individual level determinants; gender, employment status, physical activity level, and general health status were associated with depressive symptoms in our study sample. These findings reflect the role individual level factors play as probable risk or protective factors for the development of depressive symptoms in both young and adult populations. Globally, women are said to be twice likely to present with depressive symptoms than men. In 2010, global depressive symptoms prevalence estimates were 5.5% for women and 3.2% for men (45, 50), with countries like Canada registering increased prevalence levels of 5.8% in women and 3.6% in men as of 2012 (51). The observed difference in prevalence of depressive symptoms in men and women is attributed to women presenting with internalizing symptoms while in men more externalizing symptoms (52). Thus, the high prevalence of depressive symptoms in women is attributed to hormonal change specific form of depression-related illnesses which include premenstrual dysphoric, postmenopausal conditions, and postpartum stress (15). Therefore, chronic diseases worsen the occurrence of depressive episodes in women. However, the higher prevalence rate of depressive symptoms amongst Turkish women cannot be attributed to hormonal changes only. The thorough examination of the role of hormonal changes considering social demographic and other contextual variables is warranted. The complex interaction of sociodemographic factors and other personal variables, such as health status, could be ascribed to the higher rates of depressive symptoms in Turkish women. Factors such as poverty (14, 53), education (14, 16), marital status (14, 54), employment status (14, 54), and other personal traits are associated with perceived inequalities in healthcare services indicators and one’s health status, subsequently the experience of depressive symptoms. Therefore, noticeable inequalities in both social and health services indicators amongst women with low level of education, originating from rural areas, can be attributed as the cause of various health outcomes (i.e., depressive symptoms included) (16).

Furthermore, literature on the association between depressive symptoms and employment status amongst individuals with chronic diseases is bidirectional. In our study, employment status was negatively associated with episodes of depressive symptoms. These findings are consistent with a study in the USA which reported increase in depressive symptoms amongst emerging adults (18–25 years) and development of chronic conditions among unemployed individuals (17). In South Korea, loss of employment was positively associated with development and onset of both depressive symptoms and chronic conditions such as hypertension (50). Loss of employment or state of being unemployed is associated with emotional distress and financial inabilities which translate into depression and chronic conditions. On the contrary, adults with jobs are more likely to present with depressive symptoms and chronic conditions as compared to those unemployed (51). The first reason for this observation is that individuals working are likely to suffer from irregular diet due to busy schedules and hence are prone to chronic diseases (52). Chronic gastritis, shoulder and neck-pain disorders have been associated with exhaustion with busier work schedules and these trigger depressive symptoms. Another possible explanation is that working individuals are depressed due to the dual effect of physical and psychological discomfort (51). This is an outcome of pressure from their work, family, and society which generate psychological burden. Nevertheless, longitudinal population studies to examine the bidirectional relationship between depression and employment status in individuals with chronic conditions is warranted.

Results in this study further revealed a significant association between chronic conditions and depressive symptoms irrespective of the age of the participants. Globally, depression has been associated with a number of chronic morbidities such as diabetes (53), stroke (54), asthma (55), low back-pain disorders (56), Urinary incontinence (54), and many more. This association is in part evidenced to be mediated by other factors such as socio-demographics, environmental risk factors, and biological pathways (57). Developing an understanding of the complexity of the interaction between physical health and mental health status is warranted for global public health effort. This is to ensure extended quality of life and healthy life for the global population. Despite availability of extensive literature suggesting that older adults are the most commonly affected by the association between depression and chronic diseases (18, 22, 44), recently, studies have shown the relationship exists in young individuals (19, 42). Depression and chronic diseases have been bidirectionally associated. Most chronic conditions such hypertension, low back-pain, stroke, etc., are debilitating and hence lead to somatic consequences worsening the burden of depression (36). Furthermore, individuals with chronic conditions often times are faced with the need to seek healthcare services from multiple providers. This increases the likelihood of unwanted polypharmacy, exposure to effects of multiple medications, and poor adherence which are risk factors for iatrogenic complications (58). Consumption of several drugs has been reported to increase the risk of development of adverse emotional distress and mood swings, which are a recipe for depressive symptoms (59). In the elderly, the blending of multimorbidity, polypharmacy, and depression potentiates compound effects which are evidenced huge to manage than sum of its parts (36).

On the other hand, depression in adults is singled out as the most common cause of disability (60). A major consequence of depressive symptoms is the loss of an individual’s ability to perform daily task, which results in them being bidden ridden and lose various positive aspects of life (61). Depressed individuals are less likely to seek support from family and friends and have no satisfaction in the need for sense of coherence and belonging and hence become frustrated within the feeling of loneliness. Chronic conditions such as low back-pain and neck-pain disorders are known to develop from chronic depressive symptoms with other biopsychosocial factors acting as mediators of the association (45, 56). Nevertheless, increase in the prevalence of depressive symptoms in the context of physical chronic conditions have been associated with increased health costs and mortality. Further studies are therefore required to establish the cause, and develop a deeper understanding of the relationship between depression and chronic diseases.

The study had both strengths and limitations. One particular strength of our study is that we employed a much larger and well-characterized population representative sample. Our large sample size enabled us to investigate the potential associations with a better power of multivariable models adjusted for a number of possible confounders. In addition to the above, we employed as well-validated instrument in screening for depressive symptoms of the study sample. One of the principal limitations of our study was that we could only explore association and not causation between depressive symptoms and chronic conditions and individual level factors due to its cross-sectional design. Furthermore, even though the PHQ-8 has been validated and approved as a means for screening depression, the instrument does not resemble official diagnostic tools. Self-report bias was plausible in our study as all variables were based on self-reported outcomes. Nonetheless, the association between depressive symptoms and chronic diseases observed in our analyses signifies the existence of the relationship despite plausible self-report bias.

In summary, at individual level, gender and general health status were associated with increased odds of depression. Furthermore, a history of any of the chronic diseases, irrespective of age, was a positive predictor of depression in our study population. These results could be used as a basis for further research on the prevalence of the current depressive disorders and related factors in Turkey. Given the sample’s representativeness, these results can serve as a reference for monitoring depression amongst adults, planning health resources, and developing preventive and screening strategies targeting identified higher-risk population groups. Future research should focus on longitudinally evaluating the association between depressive symptoms and chronic conditions to establish causation.

## Data availability statement

The datasets presented in this study can be found in online repositories. The data used in this study was accessed from, with permission, from TSI. The data is publicly accessible upon request and registration through the website https://data.tuik.gov.tr/Search/Search?text=Turkey%20Health%20Survey.

## Ethics statement

The study protocol for the THS was approved by the chairperson of the TSI. “Regulation on Procedures and Principles Regarding Confidential Data Privacy and Data Security in the Official Statistics” was employed to ensure the privacy and confidentiality of the data from the study. These guidelines were officially published and a gazette was assented to on June 20, 2006-No.26204. All participants provided informed consent before enrollment into the study. The patients/participants provided their written informed consent to participate in this study.

## Author contributions

YP, KA, and ON designed the study and drafted the manuscript. KA, NGY, and HZA applied for the data access from Turkish Statistical Institute. YP, ON, KA, and MM conducted data analyses. ON supervised all data analyses work and reviewed all the draft versions of the manuscript. HZA, NGY, MM, and HC did literature review and provided advice in the study design and data analyses. All authors contributed to the intellectual content of the manuscript, read, and gave approval of the final manuscript.

## Abbreviations

AOR: adjusted odds ratios
CI: confidence interval
TSI: Turkish Statistical Institute
THS: Turkey health survey
EHIS: The European Health Interview Survey
PHQ-8: patient health questionnaire-8.
